# Amorphous calcium phosphate nanoparticles using adenosine triphosphate as an organic phosphorus source for promoting tendon–bone healing

**DOI:** 10.1186/s12951-021-01007-y

**Published:** 2021-09-08

**Authors:** Haoran Liao, Han-Ping Yu, Wei Song, Guangcheng Zhang, Bingqiang Lu, Ying-Jie Zhu, Weilin Yu, Yaohua He

**Affiliations:** 1 Department of Orthopedics, Shanghai Jiao Tong University Affiliated Sixth People’s Hospital, 600 Yishan Road, Shanghai, 200233 China; 2 State Key Laboratory of High Performance Ceramics and Superfine Microstructure, Shanghai Institute of Ceramics, Chinese Academy of Sciences, 1295 Dingxi Road, Shanghai, 200050 China; 3 Department of Orthopedics, Jinshan Branch of Shanghai Sixth People’s Hospital, Affiliated to Shanghai University of Medicine and Health Sciences, 147 Jiankang Road, Shanghai, 201599 China; 4 Department of Orthopedics, Shanghai Tenth People’s Hospital, Tongji University School of Medicine, 301 Middle Yanchang Road, Shanghai, 200072 China

**Keywords:** Rotator cuff tear, Tendon–bone healing, Calcium phosphate, Nanoparticles, Adenosine

## Abstract

**Background:**

Rotator cuff tear (RCT) is a common problem of the musculoskeletal system. With the advantage of promoting bone formation, calcium phosphate materials have been widely used to augment tendon-bone healing. However, only enhancing bone regeneration may be not enough for improving tendon–bone healing. Angiogenesis is another fundamental factor required for tendon–bone healing. Therefore, it’s necessary to develop a convenient and reliable method to promote osteogenesis and angiogenesis simultaneously, thereby effectively promoting tendon–bone healing.

**Methods:**

The amorphous calcium phosphate (ACP) nanoparticles with dual biological activities of osteogenesis and angiogenesis were prepared by a simple low-temperature aqueous solution method using adenosine triphosphate (ATP) as an organic phosphorus source. The activities of osteogenesis and angiogenesis and the effect on the tendon–bone healing of ACP nanoparticles were tested in vitro and in a rat model of acute RCT.

**Results:**

The ACP nanoparticles with a diameter of tens of nanometers were rich in bioactive adenosine. In vitro, we confirmed that ACP nanoparticles could enhance osteogenesis and angiogenesis. In vivo, radiological and histological evaluations demonstrated that ACP nanoparticles could enhance bone and blood vessels formation at the tendon–bone junction. Biomechanical testing showed that ACP nanoparticles improved the biomechanical strength of the tendon–bone junction and ultimately promoted tendon–bone healing of rotator cuff.

**Conclusions:**

We successfully confirmed that ACP nanoparticles could promote tendon–bone healing. ACP nanoparticles are a promising biological nanomaterial in augmenting tendon–bone healing.

**Graphic abstract:**

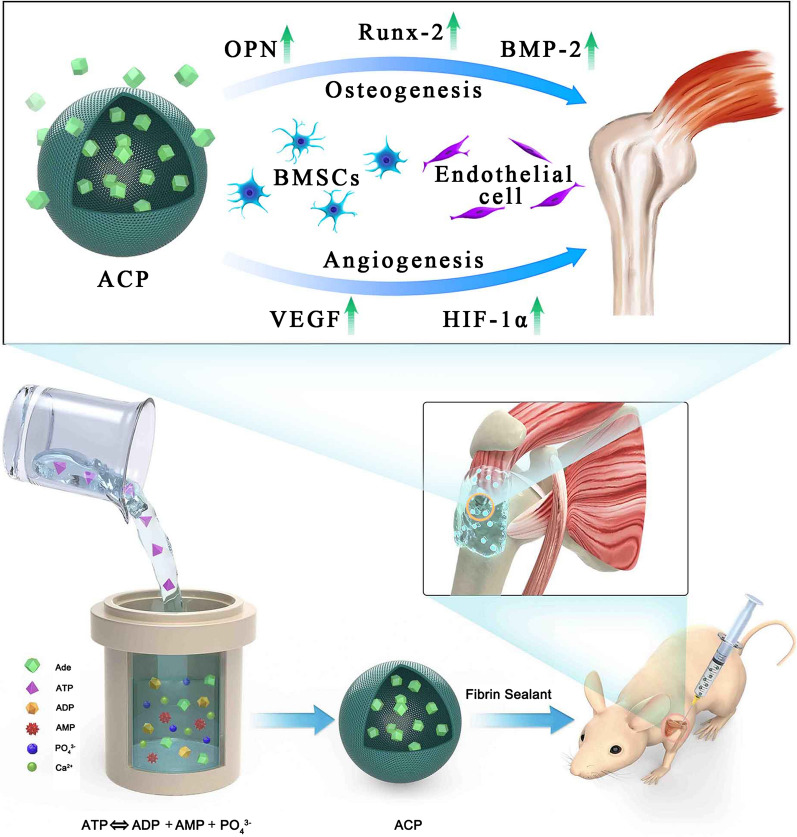

**Supplementary Information:**

The online version contains supplementary material available at 10.1186/s12951-021-01007-y.

## Background

Rotator cuff tear is a common shoulder disease, causing shoulder pain and activity limitation [1]. The prevalence of RCT was about 20.7% in general population and increases with age [2]. Although the arthroscopic rotator cuff repair (RCR) has been determined to be an effective treatment, there is still a high re-tear rate after surgery. It was reported that the re-tear rate after RCR ranged from 21% to 94% [3, 4]. Poor tendon–bone healing in the rotator cuff is believed to be the main reason for the high postoperative re-tear rate [5]. Therefore, it’s urgent to develop effective methods to promote tendon–bone healing. Normally, after rotator cuff repair, a fibrovascular tissue is formed between tendon and bone, followed by bone grows into the fibrous interface and gradually grows into the tendon, which reconstructs the continuous collagen fibers between tendon and bone. However, due to bone loss and the lack of blood vessels at the tendon–bone junction after RCR, it is difficult for bone to grow into the fibrovascular interface and tendon. Instead, the biomechanical inferior structure of scarred tissue is formed, which is prone to rerupture [6]. Hence, promoting osteogenesis and angiogenesis is essential to promoting bone growth towards the tendon–bone junction and enhancing tendon–bone healing [7–9].

Adenosine (Ade) produced in inflamed, ischemic, or hypoxic environments is able to reduce tissue injury and promote tissue repair and regeneration by involving in several receptor-mediated mechanisms [10]. Adenosine plays an indispensable role in maintaining bone homeostasis and bone regeneration [11]. Ade can act on A2bR as an autocrine/paracrine signalling molecule to enhance the osteogenic differentiation of stem cells [12]. Ade is also a signalling molecule of tissue hypoxia. Ernens et al. [13] found that ATP degradation was greater than ATP synthesis in the hypoxic tissue, leading to local aggregation of Ade in the hypoxic tissue, and Ade aggregation might play an important role in the restoration of the blood supply. It’s reported that Ade can upregulate the expression of vascular endothelial growth factor (VEGF) in cells, which might be mediated by hypoxia-inducible factor-1 (HIF-1α). Considering the dual osteogenic and angiogenic activities of Ade, we expect that Ade can be beneficial to promoting tendon–bone healing.

Calcium-phosphate based biomaterials, such as calcium-phosphate matrix and hydroxyapatite, have been widely used to promote tendon–bone healing due to their excellent biocompatibility, and osteoinductive activity [14–16]. Recently, Zhu et al. [17] successfully synthesized an organic amorphous calcium phosphate (ACP) mesoporous nanoparticles using ATP as an organic phosphorus source by the rapid microwave-assisted hydrothermal method. Compared with traditional calcium phosphate materials, ACP mesoporous nanoparticles exhibited the advantages of high stability in aqueous solution, better biocompatibility, larger specific surface area, and more stable degradation curve. Ade, the by-product of ATP hydrolysis in aqueous solution during the synthesis of ACP nanoparticles, may endow ACP nanoparticles with the dual biological activities of osteogenesis and angiogenesis [18], which make it be a suitable biomaterial for tendon–bone healing.

**Scheme 1:**
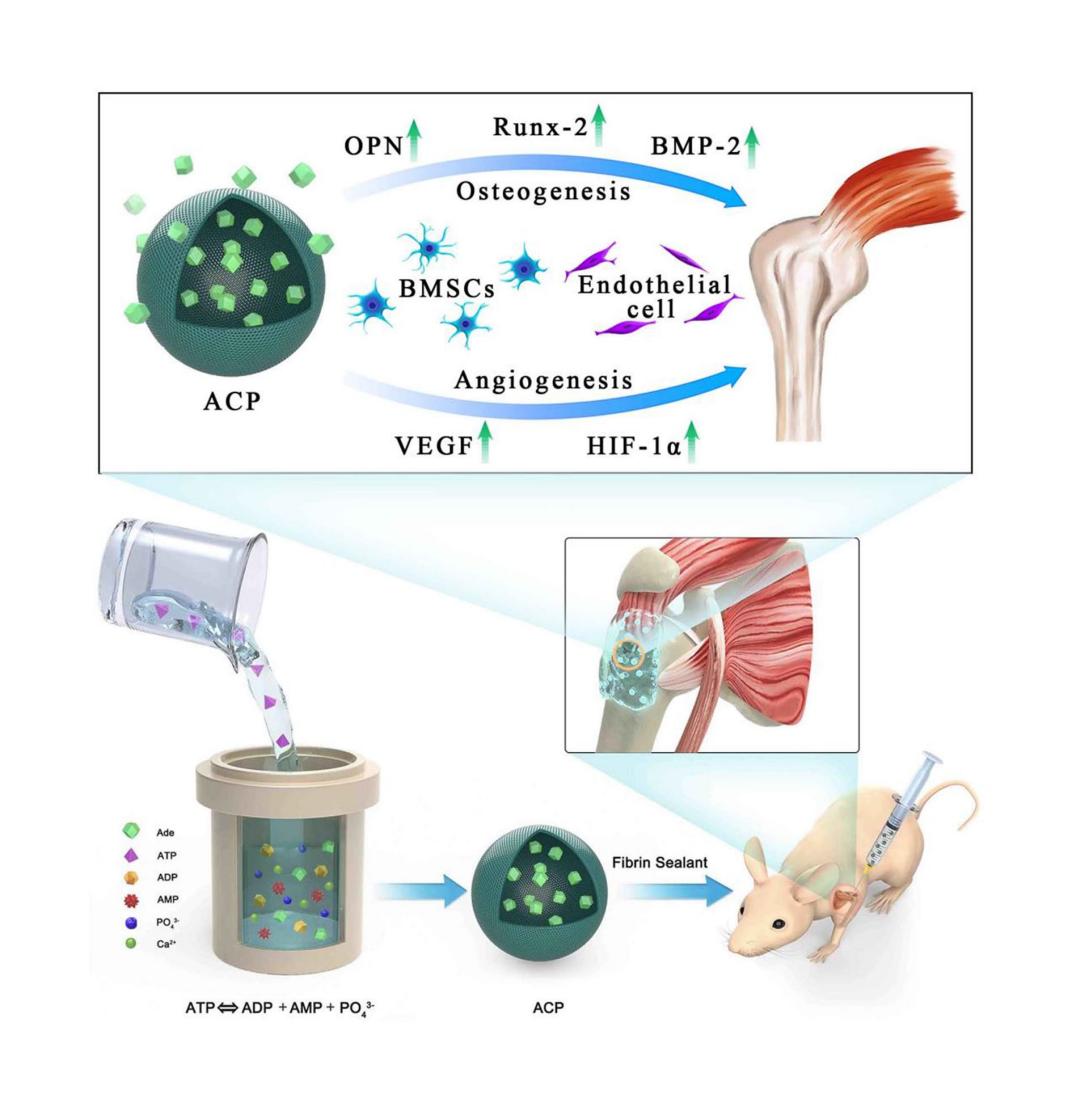
Illustration of the synthesis procedure and the promotion mechanism of amorphous calcium phosphate nanoparticles using adenosine triphosphate as an organic phosphorus source and their application in rat rotator cuff tendon–bone healing

Previous reports in ACP biomaterials mainly focus on their efficacy for bone tissue repair [19, 20], drug delivery [21, 22], and bioactive coating [23], but ACP nanoparticles for tendon–bone healing has not been reported previously. Therefore, our study aims to evaluate the efficacy of ACP nanoparticles in improving the tendon–bone healing after RCR, and we hypothesized that the ACP nanoparticles can promote tendon–bone healing due to their dual biological activities of osteogenesis and angiogenesis (Scheme 1). In vitro, the effects of ACP nanoparticles on osteogenesis and angiogenesis of hBMSCs and EA.hy926 cells were studied respectively. In vivo, the influence of ACP nanoparticles on the rotator cuff tendon–bone healing was investigated in a rat model of acute RCT.

## Materials and methods

### Preparation of ACP nanoparticles

0.555 g CaCl_2_ and 0.550 g adenosine triphosphate disodium salt (Na_2_ATP) were dissolved in 200 mL deionized water, then the pH of the solution was adjusted to 9.7 using 1 M NaOH aqueous solution. After stirring for 10 min, the resulting aqueous solution was heated at 95 ℃ for 1 h in an electric oven. After cooling to room temperature, the product was centrifuged (10,000 rpm, 3 min), washed with deionized water and ethanol twice, respectively, and then freeze dried.

For comparison, 0.555 g CaCl_2_ and 0.360 g sodium dihydrogen phosphate (NaH_2_PO_4_) were dissolved in 200 mL deionized water, then the pH of the solution was adjusted to 9.7 using 1 M NaOH aqueous solution. After stirring for 10 min, the resulting aqueous solution was heated at 95 ℃ for 1 h in an electric oven.

### Characterization of ACP nanoparticles

The samples were characterized by scanning electron microscopy (SEM, Hitachi S-4800, Japan), transmission electron microscopy (TEM, Tecnai G2 F20), X-ray diffraction (XRD, Rigaku, Ultima IV, Cu Kα radiation, *λ* = 1.54178 Å), Fourier transform infrared spectroscopy (FTIR, FTIR-7600, Lambda Scientifc, Australia), thermogravimetric (TG) analysis (STA 409/PC, Netzsch, Germany; heating rate 10 °C/min in flowing air) and surface area analyzer (Tristar II 3020, micromeritics, USA).

### In vitro experiments

#### Cell culture

All procedures were approved by the Ethical Committee of Shanghai Jiao Tong University Affiliated Sixth People’s Hospital (Approval number: 2019-KY-036(K)). We have isolated hBMSCs from 3 patients who underwent total hip arthroplasty. The experiments were repeated for 3 times. In short, suspending the aspirates in 15 mL of MEM-agar supplemented with 10% FBS (Gibco, USA) and 1% (volume/volume) penicillin/streptomycin (Gibco, USA). In this experiment, 3rd- to 5th-passage mesenchymal stem cells were used. EA.hy926 was purchased from the cell bank of the Chinese academy of sciences. The EA.hy926 is an immortalized hybridoma line that maintains the features of endothelial cells in culture. The cells were cultured at 37 °C in humidified air with 5% CO_2_.

#### Cell proliferation assay

The effects of ACP nanoparticles and Ade on the proliferation of hBMSCs and EA.hy926 cells were assessed by the CCK-8 assay (Dojindo Molecular Technologies, Inc., Japan). The method was as follows: hBMSCs or EA.hy926 cells were seeded into a 96-well plate at a density of 3 × 10^3^ cells/well. Complete medium containing different concentrations of ACP nanoparticles or Ade (0.1, 1, 10, or 100 μg/mL) was used to culture the cells for 1, 3, or 7 days. The culture medium was removed at each time point, washed twice with phosphate buffered saline (PBS), and then 100 μL of the medium with 10% CCK-8 working solution was supplemented. After incubating the cells in an incubator for 4 h, the culture medium was transferred into a 96-well plate for optical density (OD) measurement. At 450 nm wavelength, the OD of the culture medium was measured using the microplate reader (Bio-Rad 680, USA). In the following experiments, the concentration of ACP nanoparticles and Ade was 10 μg/mL.

#### Osteogenic differentiation assay

The osteogenic activities of ACP nanoparticles and Ade were evaluated by the osteogenic differentiation assay. At a density of 2 × 10^4^ cells/cm^2^, the hBMSCs were inoculated in a six-well plate and cultured in 2 mL culture medium containing ACP nanoparticles or Ade. When the cell confluence reached 60–70%, the culture medium was carefully removed, and the prewarmed complete medium for osteogenic differentiation (37 °C) was carefully added along the wall. The medium was replaced with fresh prewarmed complete medium for osteogenic differentiation every 3 days. After culturing for 21 days, the culture medium was carefully removed, and the plate was washed twice with 1 × PBS. 2 mL of 4% neutral formaldehyde solution was added to each well for 30-min fixation. Then, the neutral formaldehyde solution was aspirated, and the plate was washed with 1 × PBS twice. The Alizarin red staining solution (1 mL) was added to each well to stain cells for 5 min. After aspirating the Alizarin red, cells were washed twice with 1 × PBS and photographed.

#### Cell migration assay

The effects of ACP nanoparticles and Ade on the migration of EA.hy926 cells were assessed in a Transwell assay. EA.hy926 cells were seeded into the upper chamber of a 24-well Transwell plate (Corning; pore size = 8 μm) at a density of 5 × 10^4^ cells/well. ACP nanoparticles or Ade was added into culture medium in the lower chamber of the Transwell plate. After incubation for 24 h, the cells on the upper surface of the Transwell plate were gently wiped off with a cotton swab. The cells on the lower surface of the chamber were fixed with 4% paraformaldehyde for 10 min, stained with 0.5% crystal violet solution for 30 min, rinsed twice with PBS, and observed under an optical microscope. Finally, 500 μL of 33% acetic acid solution was used to dissolve the crystal violet adsorbed by the cells in the lower chamber, and the OD value was measured in the microplate reader at 595 nm.

#### Cell tubule formation assay

EA.hy926 cells were used to assess the angiogenic activity of ACP nanoparticles and Ade. Firstly, 50 μL of growth-factor-reduced Matrigel (Becton Dickinson, MA, USA) were added to a 96-well plate, and the plate was incubated for 30 min at 37 °C to allow the Matrigel to coagulate. EA.hy926 cells were pre-treated with ACP nanoparticles- or Ade-containing culture medium for 30 min, and then the cells at a density of 2 × 10^4^ cells/well were added to the surface of the Matrigel and incubated for 8 h in a cell incubator. Finally, the number of capillaries with a complete polygonal structure was analysed, and the tubule formation was observed under a light microscope.

#### RT-qPCR

The osteogenic and angiogenic activities of ACP nanoparticles and Ade were evaluated by RT-qPCR. The hBMSCs were cultured in the medium containing ACP nanoparticles or Ade for 7 and 14 days. Trizol reagent (American Center for Molecular Research) was used for the separation of total RNA. According to the manufacturer’s instructions, the PrimeScript™ RT Master Mix (TaKaRa, Japan) was used to perform reverse transcription of RNA to yield complementary DNA. The expression levels of OPN, OCN, BMP-2, Runx2, VEGF, TGF-β, and bFGF were detected by qPCR (MJ Research, Canada). SYBR Prexy Ex Taq TM (Takara, Japan) was used to perform quantification. The relative expression levels of these genes were normalized to GAPDH gene expression. The primer sequences used in this study are listed in Additional file 2: Table S1.

#### Western blot

The osteogenic and angiogenic activities of ACP nanoparticles and Ade were evaluated by Western blot. The hBMSCs were cultured in the medium containing ACP nanoparticles or Ade medium for 7 days or 14 days. The cells were washed with cold PBS and lysed on ice with radio-immunoprecipitation assay lysis buffer (Roche, Switzerland) containing protease inhibitor and phosphatase inhibitor for 30 min. At 4 °C, the lysate was centrifuged at 12,000 rpm for 15 min, and the supernatant in the test tube was collected. Cell lysates in equal amounts were electrophoresed and transferred onto a nitrocellulose membrane. At 4 °C, the membrane containing various proteins was incubated overnight with primary antibodies against Runx2, BMP-2, OPN, VEGF, and HIF-1α. In the Tris-buffered saline, the membrane was then washed with 0.1% Tween-20 solution three times for 10 min. The diluted secondary antibody (anti-rabbit or anti-mouse secondary antibody, 1:3000, CST, USA) was incubated with the membrane in blocking buffer at room temperature on a shaker for 1 h. Electrochemiluminescence reagents (Millipore, USA) were used to visualize immunoreactive bands.

### In vivo experiments

#### Animal surgery

Approval was obtained from the Animal Experiment Ethics Committee of Shanghai Jiao Tong University Affiliated Sixth People’s Hospital (Approval number: DWSY2019-0134). 144 male Sprague–Dawley rats weighed between 350 and 400 g underwent a bilateral supraspinatus tendon repair. The rats were anaesthetized with pentobarbital sodium (45 mg kg^−1^). A midline skin incision was made on the anterolateral shoulder, and the supraspinatus tendon was exposed after splitting the trapezius muscle. The acromioclavicular joint was cut to reveal the attachment point of the supraspinatus tendon. The insertion into the humeral great tuberosity of the supraspinatus tendon was excised, and then the enthesis was gently decorticated. The bone tunnel was formed cross the humeral head using a 0.5-mm drill head, and then the separated supraspinatus tendon was pulled out through the bone tunnel using modified Mason-Allen suture and 3–0 Ethibond suture (Ethicon) and fixed to the original footprint area. The 144 rats were divided into the following three groups randomly: (i) fibrin sealant group (FS group); (ii) Ade/fibrin sealant mixture group (Ade/FS group); and (iii) ACP nanoparticles /fibrin sealant mixture group (ACP/FS group). We implanted FS, Ade/FS, and ACP/FS into the tendon–bone junction, and then closed the wound in layers. The rats could move in cages without restriction.

#### Micro-computed tomography (CT) image analysis

Forty-eight rats were sacrificed at 4 weeks or 8 weeks after operation, every eight rats per group. Micro-CT image analysis and histological evaluation were performed. Specimens were treated with 4% paraformaldehyde for 48 h. Micro-CT (SkyScan1176, Bruker, Germany) was used to observe the bone mass density (BMD) and bone healing, especially at the beginning of the greater tuberosity. The scanning conditions were 80 kV and 450 mA. After a customized threshold scan using the selected 5 × 5 m^2^ cylindrical region of interest (ROI), the ROI was focused on the tendon–bone attachment point and covered the entire greater tuberosity. The BMD and bone volume fraction (bone volume/total volume, BV/TV) of the greater tuberosity were measured for the volume of interest (VOI) of the rotator cuff suture site.

#### Histological evaluation

Forty-eight rats were sacrificed at 4 weeks or 8 weeks after operation, every eight rats per group. Histochemical analysis was performed. The supraspinatus tendon–humerus complexes were fixed in 10% neutral buffered formalin for more than 24 h. The specimens were then calcified, dehydrated, embedded in paraffin, and cut into 5 μm—thick sections. HE, Safranin-O/fast green, picrosirius red, CD31 antibody, α-SMA antibody, type I collagen antibody, and type II collagen antibody were used for staining. To evaluate the composition of collagen, a polarizing microscope (Eclipse E800, Nikon) was used to photograph the sections stained with picrosirius red.

#### Biomechanical testing

Forty-eight rats were sacrificed at 4 weeks or 8 weeks after operation, eight rats per group. Biomechanical testing was performed. Before testing, harvested specimens was preserved in a − 80 °C freezer. The supraspinatus tendon–humerus complexes were retained, and the surrounding soft tissue was removed after thawing at room temperature. The cross-sectional area of supraspinatus tendon inserted into the humeral head was measured with a digital calliper. The samples were transferred to a custom-designed single-axis test system. The middle part of the supraspinatus tendon was fixed with screws, and the humerus was fixed with a customized vise. The specimen was preloaded to 1 N, and loaded to failure at a rate of 2 mm/s. The ultimate load-to-failure ratio was recorded. A micrometre system (1 mm resolution) connected to a linear platform was used to measure the displacement. The final load–displacement curve was used to calculate the stiffness.

### Statistical analysis

The mean ± SD was used to express all analytical results. One-way ANOVA or Student’s t-test was used to perform the statistical analysis. P < 0.05 indicates a statistically significant difference.

## Results

### Synthesis and characterization of ACP nanoparticles

**Fig. 1:**
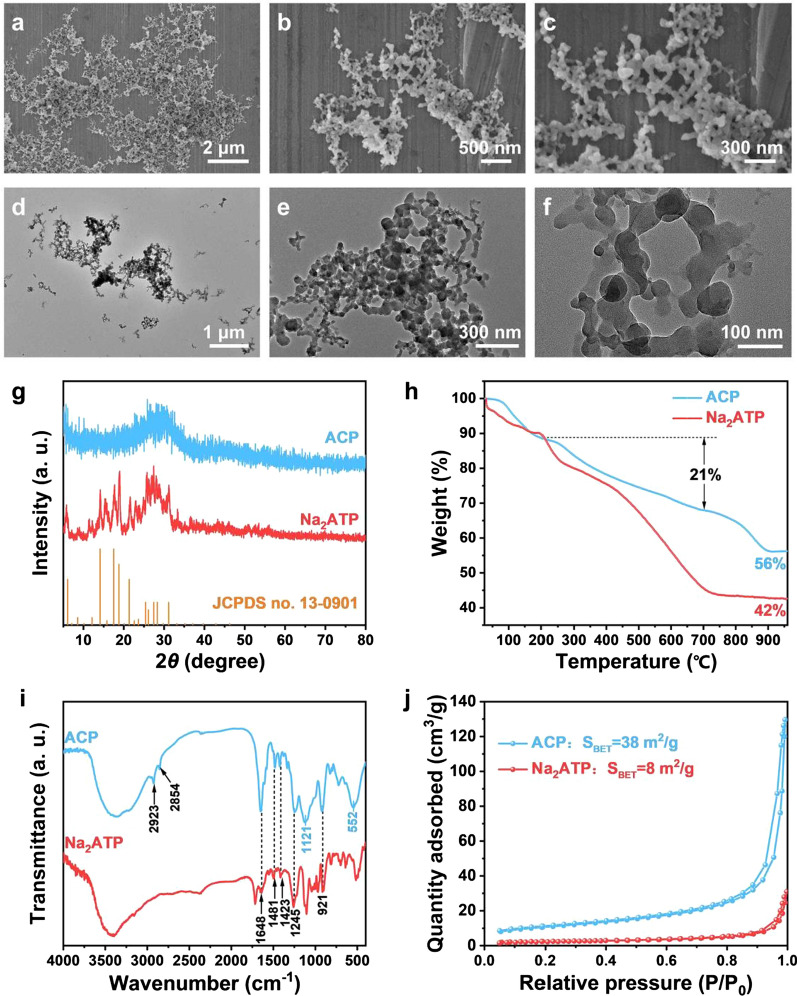
Characterization of the as-prepared ACP nanoparticles prepared using CaCl_2_ as the calcium source and ATP as the organic phosphorus source in aqueous solution at 95 ℃ for 1 h. **a**–**c** SEM images. **d**–**f** TEM images. **g** XRD patterns. **h** FTIR spectra. **i** TG curves, and **j** nitrogen adsorption–desorption isotherms

In this work, we developed a simple, rapid, environmentally friendly and scalable low-temperature solution method for the preparation of ACP nanoparticles. The ACP nanoparticles were prepared using CaCl_2_ as the calcium source and ATP as the organic phosphorus source in aqueous solution at 95 ℃ for 1 h. The morphology of the product prepared using CaCl_2_ as the calcium source and ATP as the organic phosphorus source in aqueous solution at 95 ℃ for 1 h was observed by SEM and TEM. The product consisted of solid nanoparticles with diameters of tens of nanometers (Fig. 1a–f).

The XRD pattern of the product prepared using CaCl_2_ as the calcium source and ATP as the organic phosphorus source in aqueous solution at 95 ℃ for 1 h showed a broad weak peak locating at around 2θ = 30°, suggesting the amorphous calcium phosphate (ACP) of the as-prepared product, as shown in Fig. 1g. In addition, the XRD pattern of Na_2_ATP is also shown in Fig. 1g, which exhibited a low-crystalline structure, and it was consistent with the data reported in JCPDS No. 13-0901.

FTIR spectra of the as-prepared ACP nanoparticles and Na_2_ATP are shown in Fig. 1h. The absorption peaks at 2923 and 2854 cm^−1^ originated from the –CH_3_ and –CH_2_ of ATP molecules, respectively. The strong absorption peak at 1648 cm^−1^ was ascribed to the stretching vibration mode of the –C=C– group from ATP molecules. Additionally, the absorption peaks located at 1481, 1423, 1245 and 921 cm^−1^ were attributed to ATP molecules, indicating that ATP molecules were adsorbed on the surface of the as-prepared ACP nanoparticles. Thus, the ATP molecules served as the stabilizer and could glue ACP nanoparticles together. This was also consistent with the TG analysis results. As shown in Fig. 1i, the TG analysis of ACP nanoparticles showed a weight loss of about 21% in the range of 200–700 °C, during which the decomposition of ATP molecules in ACP nanoparticles occurred. For the TG curve of Na_2_ATP, it retained 42% of original weight at 900 °C. Moreover, the BET specific surface area of the as-prepared ACP nanoparticles was measured to be 38 m^2^/g, and that of Na_2_ATP was only 8 m^2^/g (Fig. 1j). The relatively high specific surface area of ACP nanoparticles would provide more sites for cell adhesion and promote the proliferation and differentiation of the cells.

We also investigated the formation mechanism of ACP nanoparticles. As shown in Additional file 1: Fig. S1, when the pH value of the solution was adjusted to 9.7 at room temperature, ACP nanoparticles were formed, which was attribute to the ATP hydrolysis in the alkaline environment. On the contrary, when the pH value of the aqueous solution containing CaCl_2_ and Na_2_ATP was about 3, there was no product formed, indicating that ATP did not hydrolyze in this acidic environment (Additional file 1: Fig. S2a). After pH adjustment (pH 9.7) using NaOH aqueous solution, the pH value of the solution decreased with the increase of reaction temperature, and finally decreased to an acidic value (about 5.5) (Additional file 1: Fig. S2a). Interestingly, by comparing the pH change of the alkaline aqueous solution at room temperature and at 95 ℃, we found that the high temperature could accelerate the ATP hydrolysis and the formation of ACP nanoparticles (Additional file 1: Fig. S2b). Moreover, when using inorganic phosphate (NaH_2_PO_4_) as the phosphorus source, ACP nanoparticles rapidly crystallized into hexagonal hydroxyapatite phase due to the lack of ATP stabilizer (Additional file 1: Fig. S3). In a word, the alkaline environment is a precondition of ATP hydrolysis and ACP formation, and ATP molecules are the stabilizer of ACP nanoparticles, and the high temperature can accelerate the reaction rate.

### The dual biological activities of osteogenesis and angiogenesis of ACP nanoparticles

**Fig. 2:**
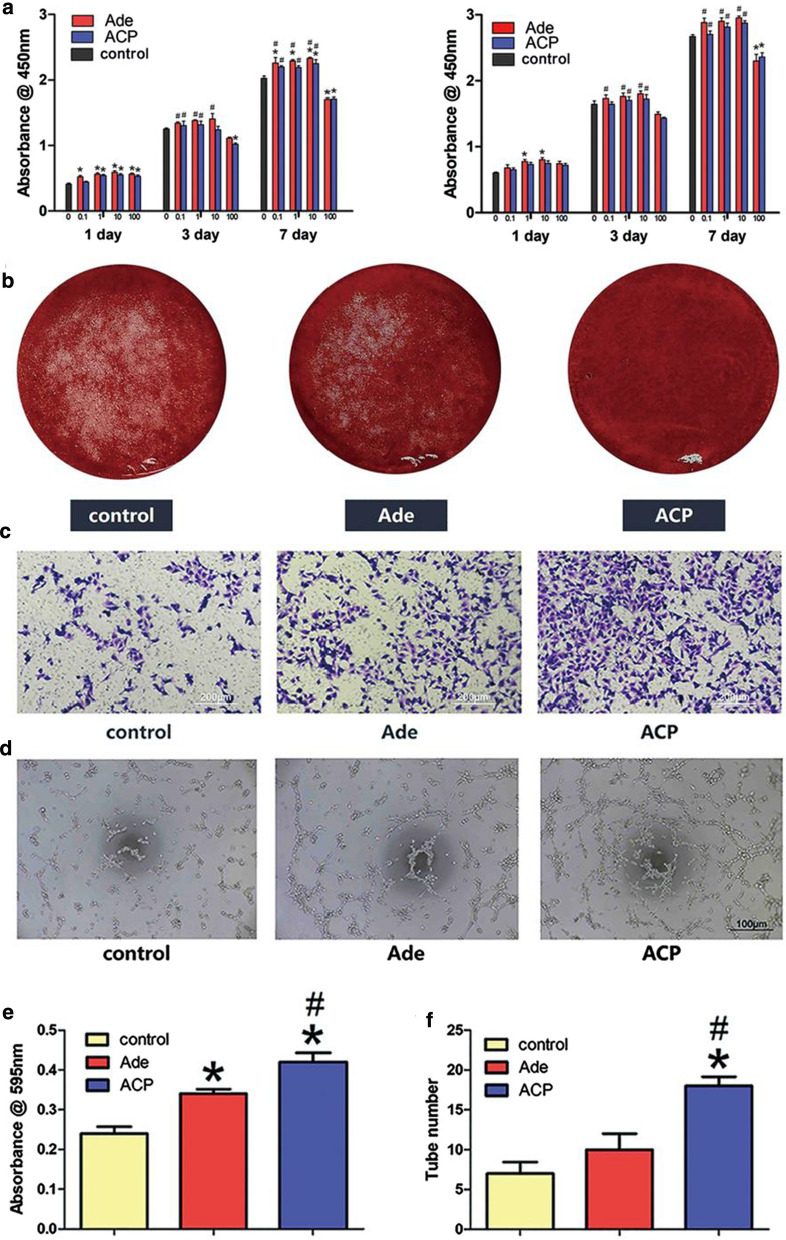
The dual biological activities of osteogenesis and angiogenesis of ACP nanoparticles. **a** The effect of Ade and ACP nanoparticles on the cell viability. (Left) Proliferation of hBMSCs; (Right) proliferation of EA.hy926 cells. **b** The effect of Ade and ACP nanoparticles on the osteogenic differentiation of hBMSCs. **c** Representative photographs showing the effects of ACP nanoparticles on the migration of EA.hy926 cells. **d** Representative photographs showing the effects of ACP nanoparticles on the tube formation of EA.hy926 cells. **e** Quantitative analysis of the migration of EA.hy926 cells. **f** Quantitative analysis of the tube formation of EA.hy926 cells (*comparison between Ade and the blank control, #comparison between Ade and ACP nanoparticles, p < 0.05)

When the concentration of Ade and ACP nanoparticles was 0.1, 1 or 10 μg/mL, hBMSCs and EA.hy926 cells was keeping proliferating at day 1, 3 and 7. When the concentration reached 100 μg/mL, the cell proliferation was inhibited at day 3 and 7 (p < 0.05) (Fig. 2a). As shown in the Fig. 2b, after culturing in osteogenic differentiation medium for 21 days, both Ade and ACP nanoparticles enhanced the osteogenic differentiation of hBMSCs, and the effect of ACP nanoparticles was strongest. ACP nanoparticles promoted the migration and tubule formation. The cells were elongated and interconnected to form a tubular structure, while the Ade-treated cells were scattered or had an incomplete tubular structure (Fig. 2c, d). Quantitative analysis showed that both Ade and ACP nanoparticles promoted the migration of EA.hy926 cells, and the promoting effect of ACP nanoparticles was strongest (Fig. 2e). Quantitative analysis showed that ACP nanoparticles significantly enhanced the tubule formation compared to Ade and the control (Fig. 2f). In conclusion, ACP nanoparticles are conducive to osteogenesis and angiogenesis.

### Effects of Ade and ACP nanoparticles on the expression levels of genes and proteins related to osteogenic and angiogenic activities in hBMSCs

**Fig. 3:**
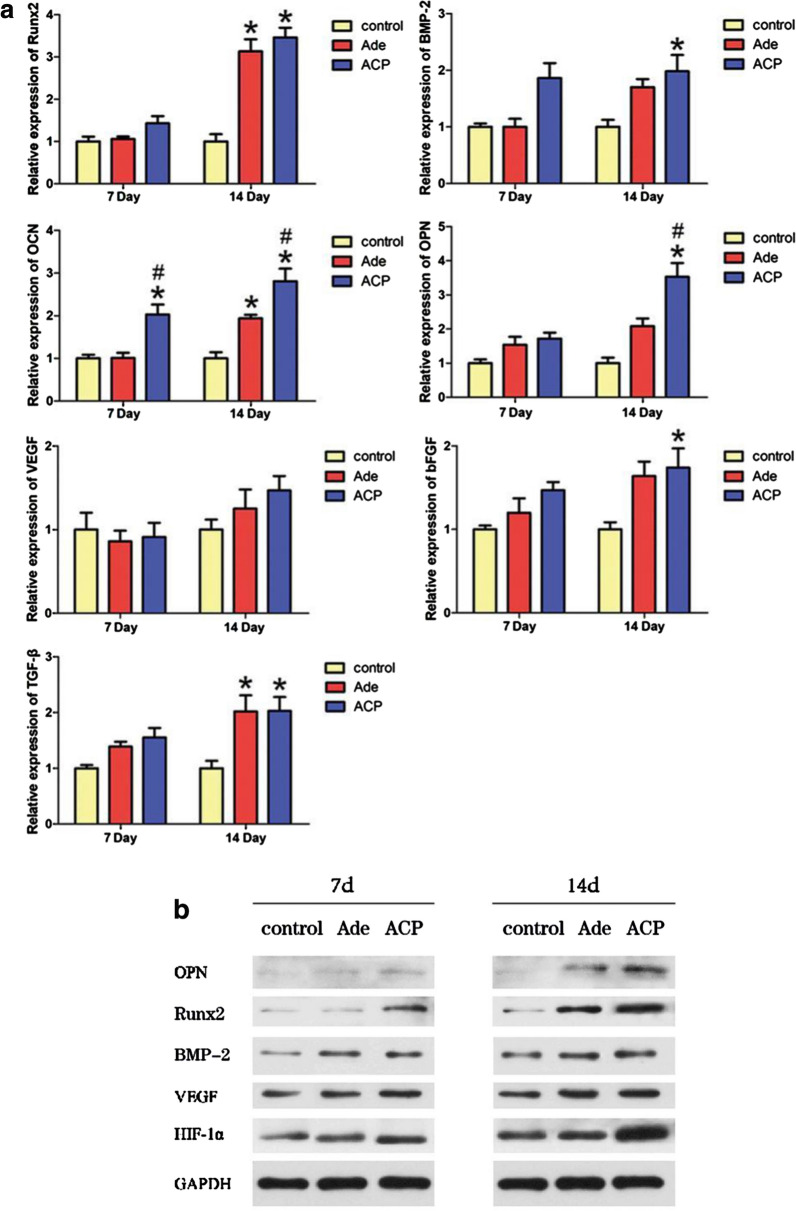
Effects of Ade and ACP nanoparticles on the expression levels of genes and proteins related to osteogenic and angiogenic activities in hBMSCs. **a** The effects of Ade and ACP nanoparticles on the expression of osteogenesis-related genes(Runx2, BMP-2, OCN, OPN) and angiogenesis-related genes(VEGF, bFGF, TGF-β) in hBMSCs (*comparison between Ade and the blank control, #comparison between Ade and ACP nanoparticles, p < 0.05). **b** Western blot analysis for the expression of OPN, Runx2, BMP-2, VEGF and HIF-1α in hBMSCs

As shown in Fig. 3a, with regards to the genes related to osteogenesis, only ACP nanoparticles upregulated the expression of OCN at day 7. At day 14, Ade and ACP nanoparticles upregulate the expression of Runx2 and OCN at the same time. However, the effect of ACP nanoparticles was strongest. Moreover, ACP nanoparticles upregulate the expression of BMP-2 and OPN. When it comes to the genes related to angiogenesis, the gene expression between three groups seems no difference at day 7. At day 14, although the expression of VEGF still shows no difference, ACP nanoparticles enhanced both the expression levels of bFGF and TGF-β and the effect of ACP nanoparticles was strongest. In the Ade-treated hBMSCs, the expression level of TGF-β was also upregulated. As shown in Fig. 3b, at day 7, both the ACP nanoparticles and Ade groups showed increased expression levels of BMP-2, VEGF and HIF-1α. Meanwhile, ACP nanoparticles also upregulated the expression of Runx-2, while the expression of OPN seems no difference. At day 14, the expression levels of OPN, HIF-1α and Runx-2 were enhanced in the ACP nanoparticles and the expression in the ACP nanoparticles group was significantly higher than that in the FS and Ade groups. The BMP-2 and VEGF expression was no difference in the all three groups.

### Micro-CT analysis

**Fig. 4:**
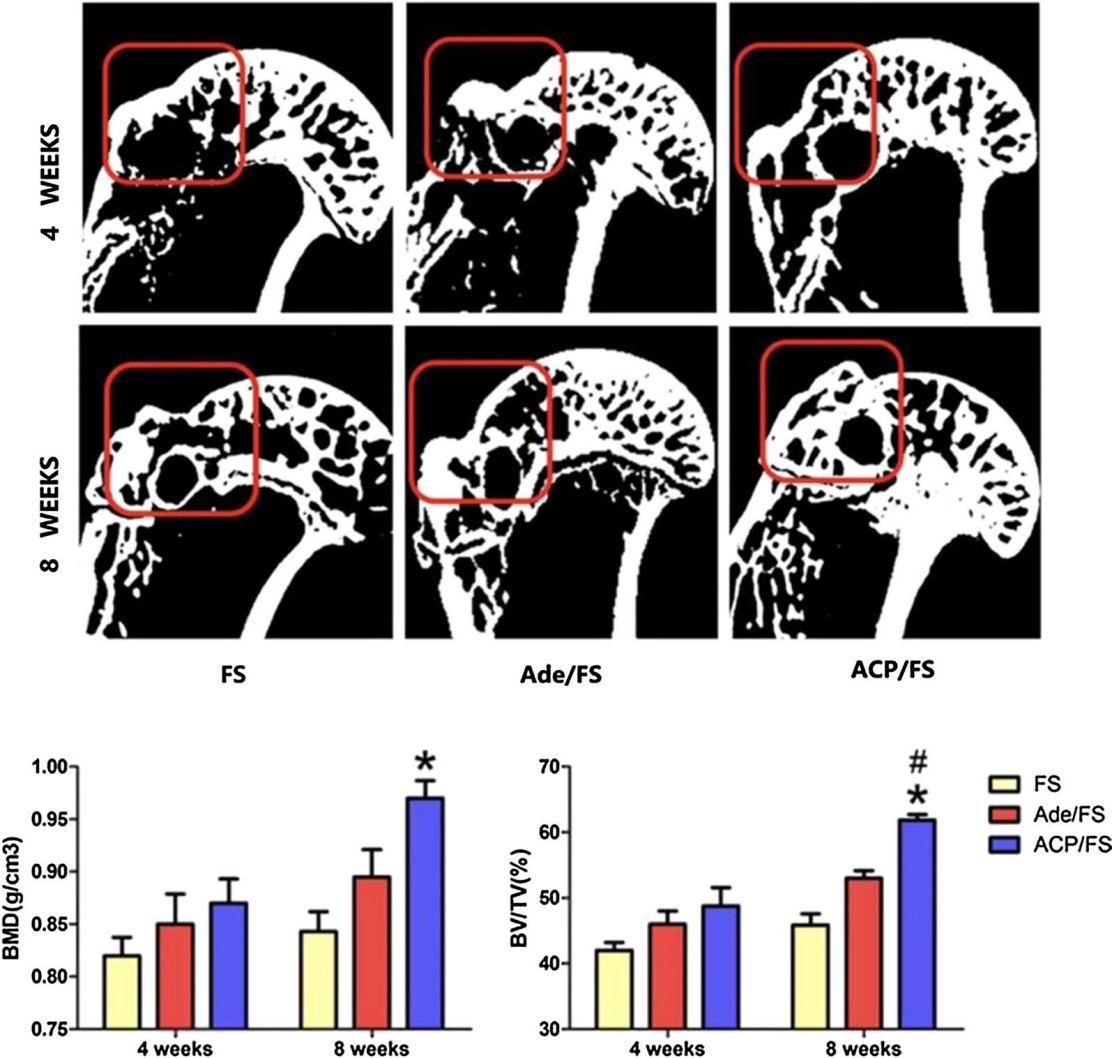
Micro-CT images showing the repair effect of the Ade/FS group and ACP/FS group for 4 and 8 weeks, respectively. Fibrin sealant group (FS group); Ade/fibrin sealant mixture group (Ade/FS group); and ACP nanoparticles /fibrin sealant mixture group (ACP/FS group) (*comparison between the Ade/FS group and FS group, #comparison between the Ade/FS group and ACP/FS group, p < 0.05)

The reconstructed micro-CT scans of the proximal humerus were used to determine the morphology of the regenerated bone (Fig. 4). When selecting the sectional view, the maximum diameter from the humeral head to the greater tuberosity was used to select the largest coronal view, and the healing of the enthesis was observed. The box at a red insertion point encircled the greater tuberosity. At the tendon–bone insertion site in the proximal humerus, the bone formation in the ACP/FS group was more prominent than those in the Ade/FS and FS groups. Micro-CT analysis showed that at week 8, the BMD in the ACP/FS group was higher than those in the FS group, and BV/TV in the ACP/FS group was higher than that in the Ade/FS and FS groups, while at week 4 and week 8, the BMD and BV/TV were not significantly different between the Ade/FS and FS groups. These results indicated that ACP nanoparticles significantly promoted new bone formation and increased the success rate of RCT healing.

### Representative HE and Safranin-O/fast green staining of the supraspinatus tendon enthesis at 4 and 8 weeks postoperatively

**Fig. 5:**
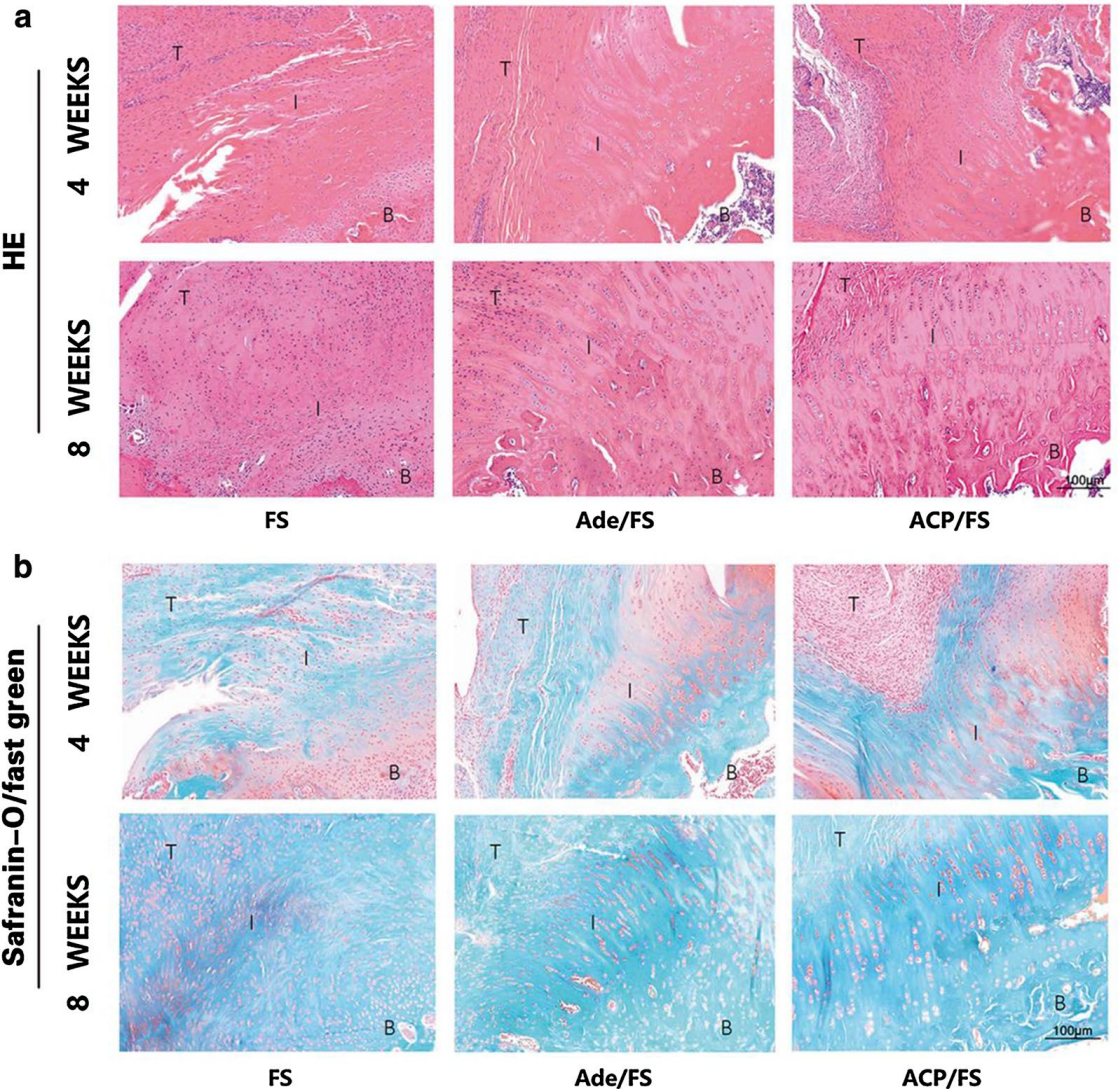
Representative HE and Safranin-O/fast green staining of the supraspinatus tendon enthesis at 4 and 8 weeks postoperatively. **a** HE staining. **b** Safranin-O/fast green staining. B, bone; I, interface; T, tendon

HE-staining showed that at week 4, there are obvious fibrovascular tissue between tendon and bone in all three groups. At week 8, there was still immature fibrovascular tissue in the tendon–bone junction. However, chondroid-like region was detectable in both tendon–bone interface in the FS group and ACP/FS group (Fig. 5a). By Safranin-O/fast green staining, the area of fibrocartilage in the ACP/FS and Ade/FS groups was significantly larger than that in the FS group, and the area of fibrocartilage in the ACP/FS group was larger than that in the Ade/FS group, indicating that the ACP/FS group had more fibrocartilage regeneration (Fig. 5b).

### Representative picrosirius red and collagen I/II staining of the supraspinatus tendon enthesis at 4 and 8 weeks postoperatively

**Fig. 6:**
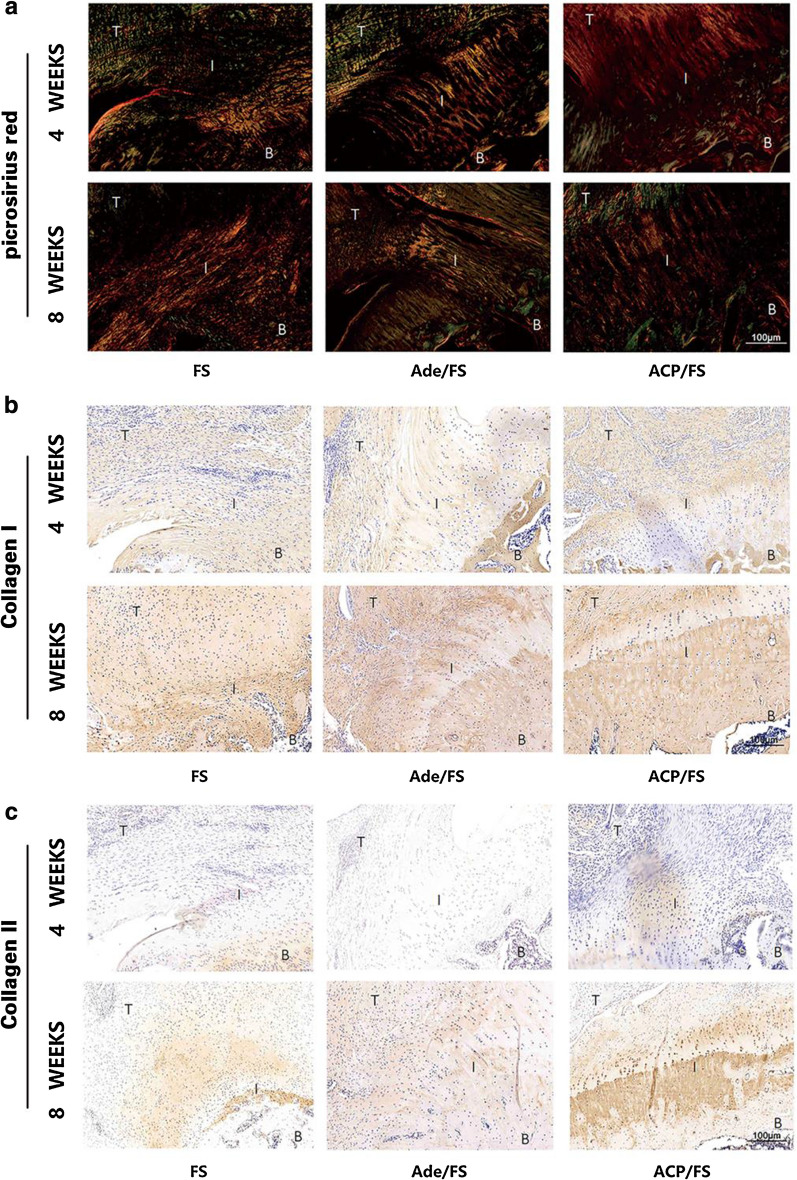
Representative picrosirius red and collagen I/II staining of the supraspinatus tendon enthesis at 4 and 8 weeks postoperatively. **a** picrosirius red staining. **b** collagen I staining. **c** collagen II staining. B, bone; I, interface; T, tendon

By picrosirius red staining, the tendon–bone interface in the ACP/FS group showed more continuous fibrous tissues with an even distribution and less variation than that in the FS group (Fig. 6a).By collagen I immunohistochemical staining, tendons in the ACP/FS group showed more type I collagen deposition than that in the Ade/FS and FS group (Fig. 6b). By collagen II immunohistochemical staining, a limited area of type II collagen was regenerated around the tendon–bone interface in the FS and Ade/FS group. In contrast, the ACP/FS group showed abundant type II collagen in the footprint area, indicating more chondrocyte extracellular matrix regeneration (Fig. 6c).

### Immunofluorescence analysis

**Fig. 7:**
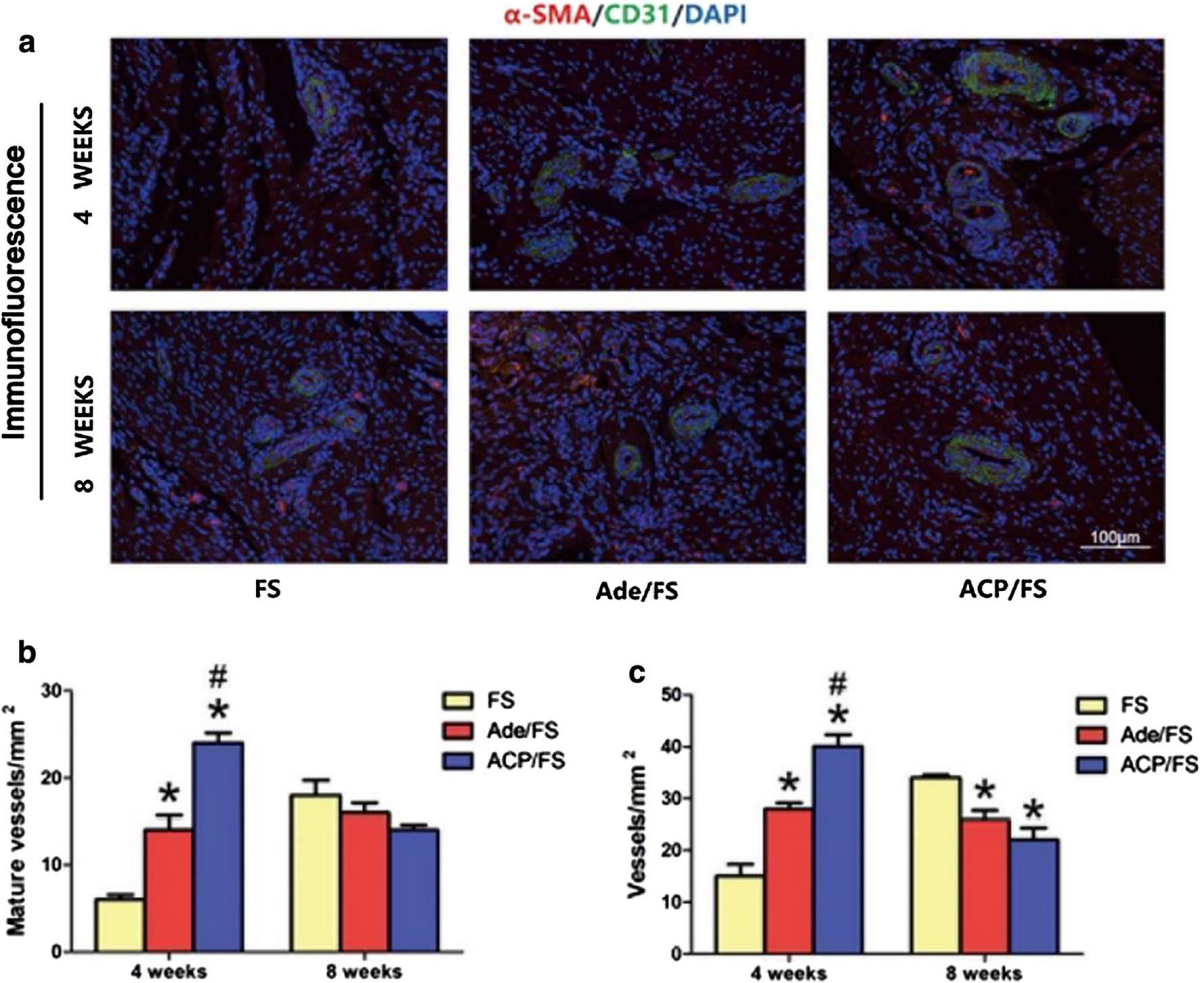
Immunofluorescence analysis. **a** Representative immunofluorescence images of α-SMA (red), CD31 (green) and DAPI (blue) stained tissue samples. **b** Quantitative analysis of the number of mature vessels in immunofluorescence images. **c** Quantitative analysis of the number of new vessels in immunofluorescence images (*comparison between the Ade/FS group and FS group, #comparison between the Ade/FS group and ACP/FS group, p < 0.05)

As for the immunofluorescence staining, at week 4, the Ade/FS and ACP/FS groups had more mature blood vessels and angiogenesis, and the ACP/FS group was better than the Ade/FS group; and at week 8, both Ade/FS and ACP/FS inhibited angiogenesis. These results indicated that ACP nanoparticles could significantly promote the formation of vessels and increase the success rate of RCR (Fig. 7a–c).

### Biomechanical testing

**Fig. 8:**
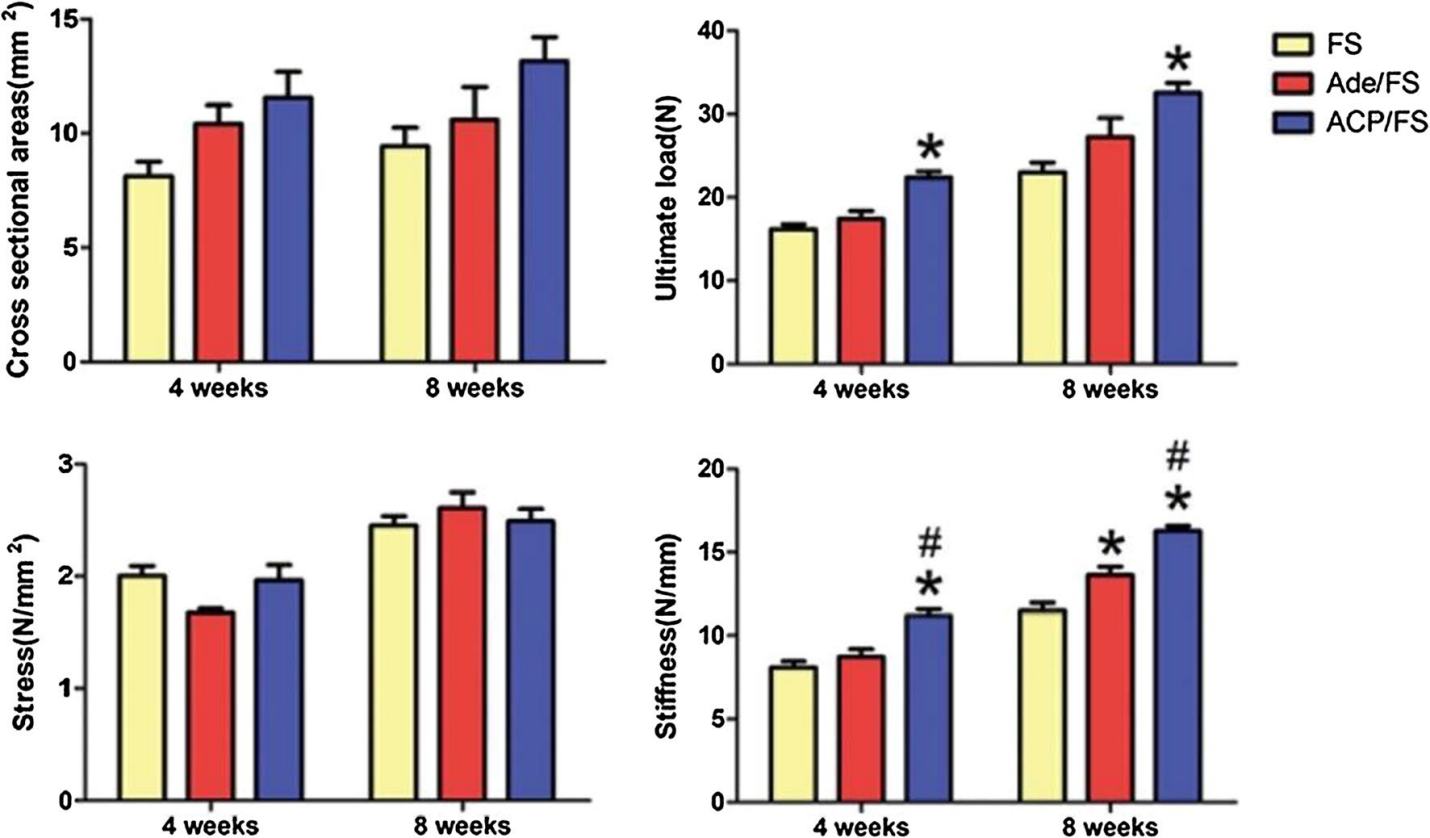
Biomechanical testing of the supraspinatus tendon-humerus complexes at the insertion site (*comparison between the Ade/FS group and FS group, #comparison between the Ade/FS group and ACP/FS group, p < 0.05)

Overall, there were no significant differences in the stress or cross-sectional area of enthesis between groups at week 4 or week 8. At week 4 and week 8, the ultimate load-to-failure and stiffness of the ACP/FS group were significantly higher than those of the FS group, and the stiffness in the ACP/FS group was significantly higher than that in the Ade/FS group. At week 8, the stiffness in the Ade/FS group was significantly greater than that in the FS group, while at week 4 and week 8, the ultimate load-to-failure was not significantly different between the Ade/FS and FS groups (Fig. 8).

## Discussion

RCT is still a challenging clinical problem for orthopedists. Due to bone loss and the lack of blood vessels at the tendon–bone junction of rotator cuff, the tendon–bone healing is mostly scar-forming healing, and the biomechanical strength of the tendon–bone junction is significantly reduced, which increases retear rate after surgical repair [24, 25]. In this study, a simple aqueous solution method was used to prepare ACP nanoparticles using biocompatible ATP as an organic phosphorus source. Adenosine, a by-product of ATP hydrolysis, could endow the ACP nanoparticles with excellent dual biological activities of osteogenesis and angiogenesis, ultimately enhancing the biomechanical properties at the tendon–bone junction and contributing to tendon–bone healing.

Promoting osteogenesis and bone growth towards the tendon–bone junction is conducive to tendon–bone healing. Inhibition of osteogenesis may damage the inward growth of bone, which may lead to anchor loosening or pull-out, eventually leading to the failure of tendon–bone healing [26]. Chung et al. [27] pointed out that osteoporosis is an independent risk factor for healing failure and has a negative impact on postoperative tendon–bone healing. In a large-scale database analysis, compared to the control group with matched age and sex and non-decreased BMD, the re-tear rate was significantly higher in patients with osteoporosis [28]. Schanda et al. [8] used zoledronic acid which is a calcium regulator in a model of rat with RCT and found that zoledronic acid provided an improvement of bone microarchitecture at the humeral head as well as an increase of ultimate failure load. Kim et al. [7] used the combination treatment of raloxifene and vitamin D in a model of rat with RCT. They found that the combination treatment could prevent a decrease in local BMD (greater tuberosity of the proximal humerus) and enhance tendon–bone healing of the rotator cuff. In this study, ACP nanoparticles show superior pro-osteogenic effects, which may be attributed to the release of adenosine. In vitro, ACP nanoparticles promoted the osteogenesis differentiation of hBMSCs. In animal experiment, micro-CT analysis showed that ACP nanoparticles significantly increased BMD and BV/TV at week 4 and week 8.

Angiogenesis is another vital factor required for tendon–bone healing [9, 29]. A sufficient blood supply is essential for the transportation of the nutrients, minerals, and oxygen required for the synthesis and mineralization of bone matrix and maturation of tendon matrix. In RCT patients, the blood supply at the tendon insertion site is interrupted, which is considered to be one of the most important reasons for incomplete healing and re-tear [30, 31]. The detection of the expression levels of CD31 and VEGF at the tendon–bone junction indicates that angiogenesis is critical for tendon–bone healing. Ye et al. [32] found that icariin can improve blood vessels formation and promote tendon–bone healing in a model of rat with RCT. In current study, the histological results showed that ACP nanoparticles promoted the postoperative expression of CD31 and VEGF and early vascular formation, which could accelerate the tendon–bone healing.

With the advantages of excellent biocompatibility and osteoinductive activity, calcium-phosphate biomaterials such as amorphous calcium phosphate and hydroxyapatite, have great potential in promoting tendon–bone healing [14–16, 33]. In an anterior cruciate ligament reconstruction model of rabbit, Tien et al. [16] used calcium phosphate cement to fill the tendon–bone interface, and they found that it could improve the biomechanical strength of rotator cuff enthesis. Mutsuzaki et al. [34] implanted calcium phosphate in the transplanted tendon tissue, and histological and mechanical examination revealed that the tendon–bone healing was accelerated. Kovacevic et al. [14] used a combination therapy with a calcium phosphate matrix and TGF-β3 in a model of rat with acute RCT and found that the combination therapy was helpful for tendon–bone healing. Huangfu et al. [35]found that a mixture of tricalcium phosphate and hydroxyapatite allowed the grafts to grow into the tunnel more quickily in a canine model. Moreover, compared with the conventionally used hydroxyapatite, repair with calcium phosphate materials interposition was better in the promotion of cell attachment and proliferation as well as new bone formation, possibly due to the increased degradability allowing for better new bone regeneration. And the Micro-CT results, histological analysis, and biomechanical testing revealed that the tendon–bone healing was promoted by calcium phosphate materials [36]. There are similar results in this study on the Micro-CT results, histological analysis, and biomechanical testing. Additionally, compared with the hydroxyapatite and the amorphous calcium phosphate, the ACP nanoparticles exhibited more advantages in biocompatibility and stability, and the ACP nanoparticles are rich in bioactive adenosine which could endow ACP nanoparticles with the dual biological activities of osteogenesis and angiogenesis. That suggests better performance for ACP nanoparticles in promoting tendon–bone healing [17, 18]. Furthermore, we adopted a simple, rapid, environmentally friendly and scalable low-temperature solution method to prepare ACP nanoparticles, which is superior to the synthetic method of traditional calcium phosphate materials. In vitro experiment, the ACP nanoparticles showed dual effects of osteogenesis and angiogenesis. In addition, histological analysis confirmed that ACP nanoparticles could effectively promoting tenon–bone integration, fibrocartilage formation, as well as tendon maturity. Biomechanical testing results showed that ACP nanoparticles enhanced the ultimate failure load and stiffness at the tendon–bone junction and thereby ultimately enhanced the biomechanical properties of the tendon–bone junction.

In this study, some limitations should be considered for future studies. For example, the repair condition was observed only at week 4 and week 8, but in clinical practice, recurrence after many years is common. In addition, only a rat model of acute RCT was established in this study, but chronic RCT is more common in clinical practice.

## Conclusion

ACP nanoparticles were prepared using biocompatible ATP as an organic phosphorus source by a simple low-temperature aqueous solution method. Ade, a by-product of ATP hydrolysis, can endow the ACP nanoparticles with excellent dual biological activities of osteogenesis and angiogenesis. ACP nanoparticles could effectively promote the formation of bone and blood vessels at the tendon–bone junction and eventually enhance the biomechanical strength of the tendon–bone junction. ACP nanoparticles are a reliable biological nanomaterial that can promote tendon–bone healing.

## Supplementary Information

**Additional file 1: **The formation mechanism of ACP nanoparticles. **Fig. S1** Characterization of ACP nanoparticles prepared using the aqueous solution containing CaCl_2_ and Na_2_ATP (pH value was adjusted to 9.7 using NaOH aqueous solution at room temperature). (a) XRD pattern. (b) FTIR spectrum. **Fig. S2** (**a**) pH change versus reaction temperature of the aqueous solution of CaCl_2_ and Na_2_ATP with (red line, pH 9.7) and without (blue line) pH adjustment using NaOH aqueous solution. **Fig. S3** (**a**) FTIR spectra of the commercial hydroxyapatite (Aladdin Industrial Corporation), the products obtained from the aqueous solution of CaCl_2_ and NaH_2_PO_4_ with pH adjustment (pH 9.7) using NaOH aqueous solution before and after heating at 95 ℃. (b) XRD pattern of the product obtained from the aqueous solution of CaCl2 and NaH2PO4 with pH adjustment (pH 9.7) using NaOH aqueous solution at room temperature.

**Additional file 2**. Primer information.

## Data Availability

All data and materials in this study are included in the published article and its additional file.

## Abbreviations

ACP: Amorphous calcium phosphate
ATP: Adenosine triphosphate
RCT: Rotator cuff tear
RCR: Rotator cuff repair
VEGF: Vascular endothelial growth factor
HIF-1α: Hypoxia inducible factor-1α
SEM: Scanning electron microscopy
TEM: Transmission electron microscopy
XRD: X-ray diffraction
FTIR: Fourier transform infrared spectroscopy
TG: Thermogravimetry
FBS: Fetal bovine serum
PBS: Phosphate buffer saline
CCK-8: Cell counting kit-8
OD: Optical density
OPN: Osteopontin
OCN: Osteocalcin
BMP-2: Bone morphogenetic protein-2
Runx2: Runt-related transcription factor 2
VEGF: Vascular endothelial growth factor
TGF-β: Transforming growth factor-β
bFGF: Basic fibroblast growth factor
BMD: Bone mass density
ROI: Region of interest
BV/TV: Bone volume/total volume
VOI: Volume of interest
