# Supplementary material for: Amorphous calcium phosphate nanoparticles using adenosine triphosphate as an organic phosphorus source for promoting tendon–bone healing
Source: J Nanobiotechnology. 2021 Sep 8;19:270. doi: 10.1186/s12951-021-01007-y (PMC8425074; doi:10.1186/s12951-021-01007-y)
Supplement: Supplementary file 1 — Additional file 1: The formation mechanism of ACP nanoparticles. Fig. S1 Characterization of ACP nanoparticles prepared using the aqueous solution containing CaCl2 and Na2ATP (pH value was adjusted to 9.7 using NaOH aqueous solution at room temperature). (a) XRD pattern. (b) FTIR spectrum. Fig. S2 (a) pH change versus reaction temperature of the aqueous solution of CaCl2 and Na2ATP with (red line, pH 9.7) and without (blue line) pH adjustment using NaOH aqueous solution. Fig. S3 (a) FTIR spectra of the commercial hydroxyapatite (Aladdin Industrial Corporation), the products obtained from the aqueous solution of CaCl2 and NaH2PO4 with pH adjustment (pH 9.7) using NaOH aqueous solution before and after heating at 95 ℃. (b) XRD pattern of the product obtained from the aqueous solution of CaCl2 and NaH2PO4 with pH adjustment (pH 9.7) using NaOH aqueous solution at room temperature. [file 12951_2021_1007_MOESM1_ESM.docx]

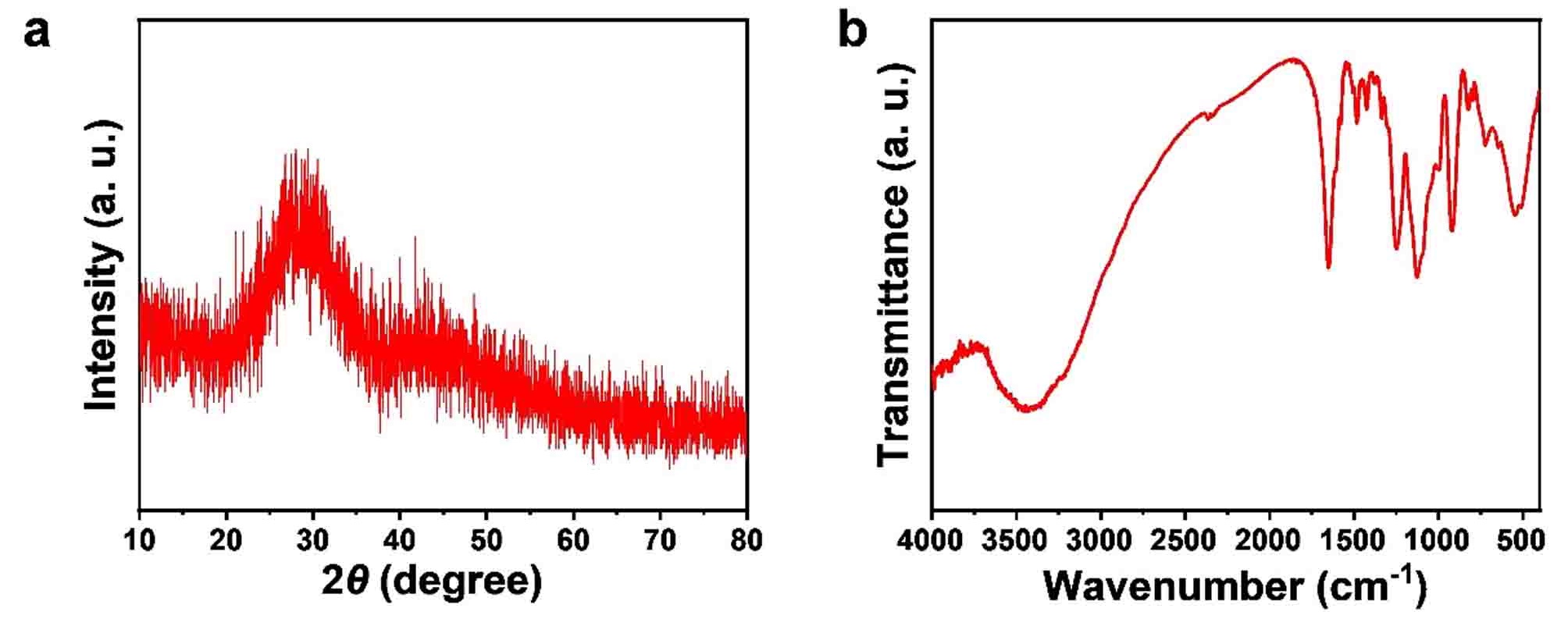


**Fig. S1** Characterization of ACP nanoparticles prepared using the aqueous solution containing CaCl_2_ and Na_2_ATP (pH value was adjusted to 9.7 using NaOH aqueous solution at room temperature). (**a**) XRD pattern. (**b**) FTIR spectrum.





**Fig. S2** (**a**) pH change versus reaction temperature of the aqueous solution of CaCl_2_ and Na_2_ATP with (red line, pH 9.7) and without (blue line) pH adjustment using NaOH aqueous solution. (**b**) pH change of the aqueous solution of CaCl_2_ and Na_2_ATP with pH adjustment (pH 9.7) using NaOH aqueous solution at room temperature and at 95 ℃.





**Fig. S3** (**a**) FTIR spectra of the commercial hydroxyapatite (Aladdin Industrial Corporation), the products obtained from the aqueous solution of CaCl_2_ and NaH_2_PO_4_ with pH adjustment (pH 9.7) using NaOH aqueous solution before and after heating at 95 ℃. (**b**) XRD pattern of the product obtained from the aqueous solution of CaCl_2_ and NaH_2_PO_4_ with pH adjustment (pH 9.7) using NaOH aqueous solution at room temperature.
